# Salidroside Delays Cellular Senescence by Stimulating Mitochondrial Biogenesis Partly through a miR-22/SIRT-1 Pathway

**DOI:** 10.1155/2019/5276096

**Published:** 2019-09-12

**Authors:** Gen-Xiang Mao, Xiao-Gang Xu, San-Ying Wang, Hui-Fen Li, Jing Zhang, Zhong-Shan Zhang, Hui-Li Su, Sha-Sha Chen, Wen-Min Xing, Ya-Zhen Wang, Ji-Huan Dai, Guo-Fu Wang, Sean X. Leng, Jing Yan

**Affiliations:** ^1^Zhejiang Provincial Key Lab of Geriatrics & Geriatrics Institute of Zhejiang Province, Department of Geriatrics, Zhejiang Hospital, Hangzhou 310013, China; ^2^Division of Geriatric Medicine and Gerontology, Department of Medicine, Johns Hopkins University School of Medicine, Baltimore, MD 21224, USA; ^3^Department of Pharmacology, Huzhou University, Huzhou 313000, China

## Abstract

Calorie restriction (CR) is a nongenetic intervention with a robust effect on delaying aging in mammals and other organisms. A mild stimulation on mitochondrial biogenesis induced by CR seems to be an important action mode for its benefits. Here, we reported that a component isolated from *Rhodiola rosea L*., salidroside, delays replicative senescence in human fibroblasts, which is related to its stimulation on mitochondrial biogenesis by activating SIRT1 partly resulted from inhibition on miR-22. Salidroside increased the mitochondrial mass that accompanied an increment of the key regulators of mitochondrial biogenesis including PGC-1*α*, NRF-1, and TFAM and reversed the mitochondrial dysfunction in presenescent 50PD cells, showing a comparable effect to that of resveratrol. SIRT1 is involved in the inducement of mitochondrial biogenesis by salidroside. The declined expression of SIRT1 in 50PD cells compared with the young 30PD cells was prevented upon salidroside treatment. In addition, pretreatment of EX-527, a selective SIRT1 inhibitor, could block the increased mitochondrial mass and decreased ROS production induced by salidroside in 50PD cells, resulting in an accelerated cellular senescence. We further found that salidroside reversed the elevated miR-22 expression in presenescent cells according to a miRNA array analysis and a subsequent qPCR validation. Enforced miR-22 expression by using a Pre-miR-22 lentiviral construct induced the young fibroblasts (30PD) into a senescence state, accompanied with increased senescence-related molecules including p53, p21, p16, and decreased SIRT1 expression, a known target of miR-22. However, salidroside could partly impede the senescence progression induced by lenti-Pre-miR-22. Taken together, our data suggest that salidroside delays replicative senescence by stimulating mitochondrial biogenesis partly through a miR22/SIRT1 pathway, which enriches our current knowledge of a salidroside-mediated postpone senility effect and provides a new perspective on the antidecrepitude function of this naturally occurring compound in animals and humans.

## 1. Introduction

Aging is the most significant risk factor for a range of degenerative disorders. One of the earlier molecular theories of aging is that reactive oxygen species (ROS) damage macromolecules progressively over time, leading to a gradual decline in cellular function [1]. ROS are mainly produced as by-products of electron transport for ATP generation by mitochondria, the cells' power plant for energy generation. Calorie restriction (CR) is arguably the most robust, nongenetic intervention that increases lifespan and reduces the rate of aging in mammals and other organisms. CR has been demonstrated to delay aging as well as the progression of age-associated disorders such as Alzheimer's disease (AD) and diabetes [2–4]. The underlying mechanisms for the beneficial effects of CR remain unknown and likely involve many processes. For instance, substantial evidence supports that CR reduces oxidative stress via stimulating the mitochondrial biogenesis. Mitochondria under CR conditions have less oxygen consumption and generate less ROS than controls. Many investigators have observed that resveratrol (RES), a SIRT1 activator, closely mimics the effects of CR. In addition, resveratrol has been shown to stimulate mitochondrial biogenesis and induce amelioration of oxidative stress [5–8]. Interestingly, resveratrol was reported to delay cellular senescence in cultured human fibroblasts [9, 10]. Therefore, developing therapeutics to improve mitochondrial biogenesis and/or its function is an attractive strategy to delay aging and prevent age-associated diseases [11]. However, difficulties and controversies are abundant in applying CR and resveratrol treatment in humans. Novel strategies to potentially induce mitochondrial biogenesis and delay cellular senescence should be explored.

Though the precise reason for the decrease in the rate of mitochondrial biogenesis during aging is currently unknown, it seems that both extra- and intracellular regulatory factors of mitochondrial biogenesis are implicated [11]. Despite the complexity of the various signaling pathways that converge to regulate mitochondrial biogenesis, they all seem to share the common key component of the peroxisome proliferator-activated receptor *γ* coactivator 1 (PGC-1) family of cotranscription factors. Specifically, PGC-1*α* has been shown to act as a common intracellular mediator during mitochondrial biogenesis [12]. PGC-1*α* enhances the expression of nuclear respiration factors (NRF-1 and NRF-2) and mitochondrial transcription factor A (TFAM), which are transcription factors that trigger the expression of genes coding for both nuclear subunits of the respiratory chain and proteins involved in mitochondrial DNA transcription and replication [13, 14]. Aging-associated reduction in mitochondrial biogenesis in aged animals shown by lower gene expression in the PGC-1*α* signaling pathways including decreased mRNA and protein contents for PGC-1*α*, NRF1/2, and TFAM accompanied with an increased oxidative stress level was observed when compared to the young controls [15]. In mammalian studies of CR or resveratrol treatment, an activation of SIRT1 was observed that resulted in deacetylation of PGC-1*α* in a nicotinamide adenine dinucleotide- (NAD-) dependent manner, leading to PGC-1*α* activation and CR- or resveratrol-induced mitochondrial biogenesis [6].

MicroRNAs (miRNAs) represent a class of naturally occurring small, 18- to 28-nucleotide-long, noncoding RNAs that negatively regulate the stability and translation of target protein-coding mRNAs at the 3′ untranslated region (UTR). Recently, accumulating evidences suggest some miRNAs, such as let-7, miR-34a, and miR-22, can modulate senescence progression by regulating the expression of senescence-related molecules such as p53 and SIRT1 [16–19].

Salidroside (SAL), a phenylpropanoid glycoside isolated from *Rhodiola rosea L*., is a popular medicinal plant used in traditional Chinese medicine. It is reputed to improve depression, enhance work performance, eliminate fatigue, and treat symptoms of asthenia subsequent to intense physical and psychological stress. Our previous studies have indicated that SAL showed a potent antiaging effect in an accelerated mouse aging model induced by D-galactose [20]. It also protects human fibroblast 2BS cells from premature senescence induced by H_2_O_2_ or UVB exposure in human diploid fibroblasts [21, 22], identified as stress-induced premature senescence (SIPS), which is mainly caused by oxidative stress and subsequent DNA damage. Besides, we found that SAL delays the replicative senescence of human diploid fibroblasts 2BS cells, which may be related to its stimulatory role on mitochondrial biogenesis. In the current study, the stimulatory effect of SAL on mitochondrial biogenesis will be investigated in 2BS cells as well as its related molecular mechanisms. We will attempt to ascertain the precise mechanisms of SAL regarding its antiaging effect *in vitro* beyond oxidative stress or DNA damage and thus inspire a new idea for prevention or delay of aging and age-associated diseases.

## 2. Materials and Methods

### 2.1. Reagents

Salidroside (SAL) was purchased from the National Institute for the Control of Pharmaceutical and Biological Products (Beijing, China). Resveratrol (3,5,4′-trihydroxystilbene), H_2_DCFDA (2′,7′-dichlorodihydrofluorecein diacetate), NAO (nonyl acridine orange), and JC-1 (5,5′,6,6′-tetrachloro-1,1′,3,3′-tetraethyl benzimidazole carbocyanine iodide) were purchased from Sigma-Aldrich. Selisistat (EX-527) was from MedChemExpress (MCE). Dulbecco's modified Eagle's medium (DMEM), fetal bovine serum (FBS), and trypsin were obtained from Invitrogen, USA. Primary anti-PGC-1*α* antibody was obtained from Novus Biologicals, LLC, USA. Antibodies for SIRT1, Rb, p21^Waf1^, and p16^INK4a^ were from Cell Signaling Technology, Inc. Anti-NFR-1 and anti-TFAM antibodies were Abcam products. Anti-p53 and anti-*β*-actin primary antibodies were purchased from Santa Cruz Biotechnology, Inc., USA.

### 2.2. Cell Culture

The 2BS cell line isolated from human fetal lung fibroblasts was originally established by the National Institute of Biological Products (Beijing, China) and has been well characterized and widely used as a cellular senescence model [23–25]. Cells are considered to be young at earlier than 30 population doubling (PD) and replicative senescent around 55PD or later. The cells were grown in DMEM supplemented with 10% FBS, 100 U/mL penicillin, and 100 mg/mL streptomycin in an incubator at 37°C with 5% CO_2_. The cultured cells were split in ratios of 1 : 2 or 1 : 4 when the confluence of the culture was over 85%. The cumulative population doublings (CPDs) were calculated as log_2_ (*D*/*D*_0_), where *D* and *D*_0_ are defined as the density of cells at the time of harvesting and seeding, respectively. As replicative senescent fibroblasts are hard to harvest for various detections, all experiments were performed using cells that were at 50PD as near-senescence (presenescence) cells unless indicated otherwise.

### 2.3. Senescence-Associated *β*-Galactosidase (SA-*β*-gal) Staining

The activity of senescence-associated *β*-galactosidase (at pH 6) is a biomarker of replicative senescence first reported by Dimri et al. [26]. The percentage of SA-*β*-gal-positive cells out of the total number of cells was counted. Average percentages were obtained from three independent experiments.

### 2.4. Intracellular Reactive Oxygen Species (ROS) Measurement

The intracellular ROS level was determined by flow cytometry analysis. Before submitting for flow cytometry analysis, cells were trypsinized and collected for an incubation with the ROS probe 2′,7′-dichlorodihydrofluorecein diacetate (H_2_DCFDA, 10 *μ*M) in the dark. The intracellular fluorescence (FL1, green) intensity was measured, and a relative level (set control as 100%) was calculated. Images of cells stained by H_2_DCFDA were acquired by Leica fluorescence microscopy.

### 2.5. Measurement of a Mitochondrial Mass

A mitochondrial mass was determined by using the nonyl acridine orange (NAO) probe, which specifically binds to cardiolipin, a specific phospholipid that is found almost exclusively in the inner mitochondrial membrane and is widely considered as a mitochondrial mass marker [3]. For flow cytometry assay, cells were treated as indicated above, detached and fixed with 70% ethanol, and stored until use at -20°C. Then, ethanol was removed by centrifugation, and cells were washed in PBS and stained with 10 *μ*M NAO in 1 mL of PBS. After incubating for 10-20 min at room temperature in the dark, cells were washed in PBS for three times and submitted for flow analysis. Relative change in mean fluorescence for green fluorescence at 530 nm was calculated which reflects the mitochondrial mass.

### 2.6. Measurement of Adenosine Triphosphate (ATP)

Intracellular ATP was measured by using the ATP colorimetric/fluorometric assay kit supplied by Abcam (ab83355). Briefly, cells rinsed by cold PBS were lysed in 100 *μ*L of ATP assay buffer and centrifuged at 15,000g for 2 min. The supernatant was collected and transferred to a new tube, and then, 20-40 *μ*L of 4 M perchloric acid were added to remove proteins by centrifugation at 12,000g for 2 min. The supernatant was collected, and a buffer containing 2 M KOH was added to neutralize the sample and remove the excess perchloric acid. After centrifugation for 15 min at 12,000g to pellet insoluble materials, the supernatant was added to a 96-well plate and developed according to the manufacturer's instructions. The absorbance was read at 570 nm using an automatic ELISA plate reader. The ATP content was calculated based on a standard curve generated at the same time.

### 2.7. Flow Cytometric Analysis of Mitochondrial Membrane Potential (*ΔΨ*m)

Changes in the *ΔΨ*m were analyzed by using JC-1. JC-1 is a positively charged fluorescent compound which is taken up by mitochondria proportionally to the inner mitochondrial membrane potential [27]. *ΔΨ*m was determined by the ratiometric analysis of orange fluorescence emitted by JC-1 aggregates (FL2, red) and that emitted by the free probe (FL1, green). Briefly, 2BS cells seeded in six-well plates were treated with SAL (10 *μ*M) for indicated time. Then, cells were dissociated and were incubated in 1 mL of PBS supplemented with 10% FBS containing 0.5 *μ*g/mL JC-1 for 1 h in the dark. Cells were then washed twice with PBS and submitted for flow cytometric analysis. The ratio of FL2 versus FL1 reflects the level of *ΔΨ*m. Images of JC-1 staining were acquired using Leica fluorescence microscopy.

### 2.8. Western Blot Analysis

Cells were rinsed with iced PBS and then lysed with cell lysis buffer containing protease inhibitor cocktail (Cell Signaling Technology, USA). Protein concentrations were determined by the BCA protein assay kit (Pierce Chemical Co.). A total of 50 *μ*g of protein extracts were loaded and electrophoresed on a 4-15% SDS polyacrylamide gel and transferred to the polyvinylidene fluoride (PVDF) membrane (Bio-Rad, USA). The membranes were subsequently probed with specific primary antibodies. The secondary antibody used for detection was linked with horseradish peroxidase. The enhanced chemiluminescence (ECL) method was used to detect the conjugated horseradish peroxidase. The optical density (OD) of each band was measured using ImageJ for semiquantitative analysis.

### 2.9. miRNA Microarray

Total RNAs were harvested from 2BS fibroblasts at different PD with or without SAL treatment by using the traditional acid guanidinium-phenol-chloroform extraction method and quantified with a spectrophotometer (NanoDrop; Thermo Fisher Scientific). Microarray analysis of the miRNA expression (miRbase 19.0) was performed by using a service provider (LC Science).

### 2.10. Real-Time qPCR

Total RNA was extracted from the cells and tissues using the miRNeasy Mini kit (QIAGEN). The expression of mature miR-22 was quantified according to a kit (Hs_miR-22_1 miScript Primer Assay targets miRNA: hsa-miR-22-3p, QIAGEN, MS00003220) by using a real-time PCR system LightCycler 480 (Roche). The expression of miRNA was defined from the threshold cycle, and relative expression levels were calculated using the 2^-*ΔΔ*Ct^ method after normalization with reference to the expression of U6 small nuclear RNA (QIAGEN, MS00033740).

### 2.11. Lentivirus Infection

Lentivirus expressing Pre-miR-22 was generated with LV3-GFP lentiviral vectors (Lenti-Pre22) provided by GenePharma Inc. (Shanghai, China). For pilot studies, cells were infected with control vector (Lenti-C) or Lenti-Pre22 at various multiplicities of infection (MOI) accompanied with 1-5 *μ*g/mL polybrene, and a MOI of 5 with 2 *μ*g/mL polybrene was optimal to transfect the 2BS cells.

### 2.12. Data Analysis

All experiments were repeated in triplicate. The results were expressed as mean ± SD. One-way ANOVA analysis (SPSS 19.0) was used for data comparisons within multiple groups, with *P* value less than 0.05 considered to be statistically significant.

## 3. Results

### 3.1. SAL Delays Replicative Senescence of Human 2BS Fibroblasts

**Figure 1:**
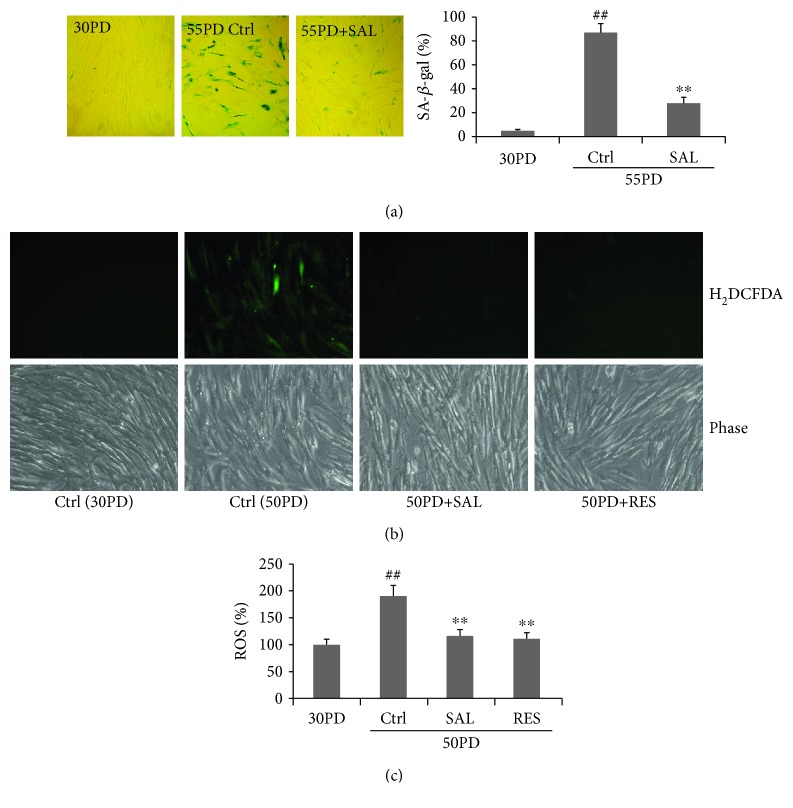
Salidroside (SAL) delays the phenotypes of replicative senescence of human fibroblast 2BS cells. (a) SA-*β*-gal staining of 2BS fibroblasts grown from 30PD in DMEM supplemented with 10 *μ*M salidroside (SAL). Control cells at 55PD became replicative senescence. Cells at 30PD were set as young control. Cells of a nonconfluent state were washed with PBS, fixed with 3% formaldehyde, and submitted for SA-*β*-gal staining. Cells were microphotographed at a magnification of 10 × 10. (b, c) Effect of SAL and resveratrol (RES) on the ROS level in near-senescent 50PD 2BS cells. Cells treated by SAL (10 *μ*M) or RES (10 *μ*M) for 24 h were stained by H_2_DCFDA for detection of intracellular ROS production. (b) Images of cells stained by H_2_DCFDA were obtained by using fluorescence microscopy. (c) Quantitative measurement of an intracellular ROS level was performed by flow cytometric analysis. ^##^*P* < 0.01 versus 30PD control (Ctrl); ^∗∗^*P* < 0.01 versus control (Ctrl) at 55PD or 50PD.

Our previous studies indicated that the optimal dose range of SAL for its protection against stress-induced premature senescence (SIPS) in human diploid 2BS fibroblasts is 5-20 *μ*M [21, 22]. In this study, we first observed the effects of SAL on replicative lifespan and biomarkers related to cellular senescence in human 2BS fibroblasts. Cells were cultured in a SAL-supplemented medium from 30PD until they became replicative senescent. SAL significantly delayed replicative senescence of 2BS cells by at least 8 PDs (see Table 1). The two concentrations of SAL (5 *μ*M and 10 *μ*M) showed a similar gain in PDs. The growth rate of SAL-treated cells was dramatically increased compared to that of the control cells (Table 1). For the SA-*β*-gal activity, a biomarker of cellular senescence, only sporadic SA-*β*-gal-positive cells were observed in young control cells (Figure 1(a)). As anticipated, SA-*β*-gal activity was markedly elevated in 55PD control cells (91.7 ± 7.1%), while cells at 55PD cultured in a 10 *μ*M SAL-supplemented medium from 30PD showed a much lower positive rate (28.3 ± 4.9%). Moreover, SAL treatment for 48 h significantly suppressed the elevated production of intracellular ROS in near-senescent 2BS cells (50PD), which was comparable to that of resveratrol (RES) (Figure 1(b)), an antioxidant reported to be a SIRT1 activator and delays replicative senescence in human fibroblasts [9]. The above results show that SAL could delay the replicative senescence process of 2BS cells.

### 3.2. SAL Induces an Increment of Mitochondrial Biogenesis in 2BS Fibroblasts

**Figure 2:**
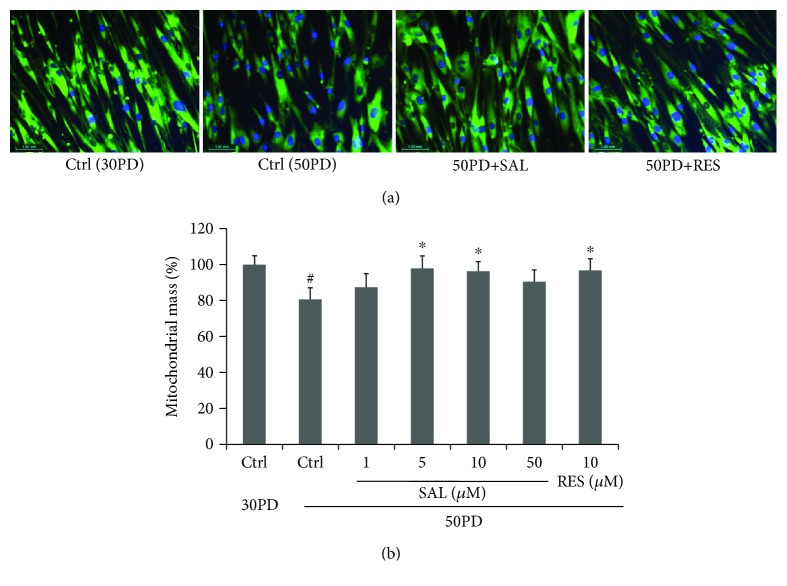
Effect of salidroside (SAL) on a mitochondrial mass. (a) Mitochondrial staining in 2BS cells incubated with SAL (10 *μ*M) or resveratrol (RES) at 10 *μ*M for 48 h. NAO signal in cells was visualized by confocal microscopy. Images were acquired by using a ×20 objective. DNA was visualized by DAPI. Pictures were shown as merged by two paired images from NAO staining and DAPI staining, respectively. (b) Mitochondrial mass in cells cultured after 48 h with SAL at different concentrations was quantified by flow cytometry by using NAO as indicated in Materials and Methods. Data indicate the relative mean of the MFI (mean fluorescence intensity) from three different experiments performed in duplicate. ^#^*P* < 0.05 versus 30PD control (Ctrl); ^∗^*P* < 0.05 versus control (Ctrl) at 50PD.

**Figure 3:**
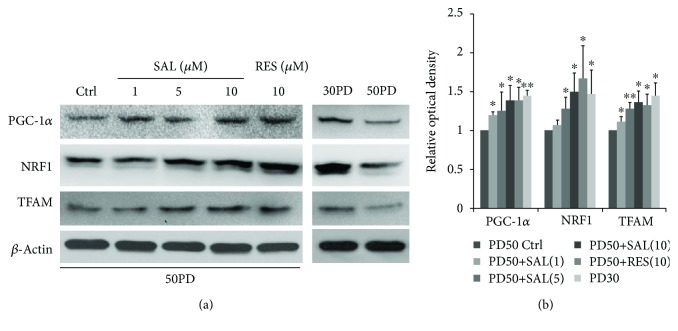
Effect of salidroside (SAL) on the mitochondrial biogenesis-associated PGC1*α*-TFAM signaling pathway. 2BS cells at 50PD were treated by salidroside (SAL) at various dosages or resveratrol (RES) at 10 *μ*M for 24 h and then were harvested for western blot analysis. (a) Representative images were acquired from three different experiments. (b) Quantitative analysis of the protein levels of PGC-1*α*, NRF1, and TFAM. Bars represent relative protein levels counted as *D*_1_/*D*_0_ (the value for 50PD control was set as 1.0), where *D*_0_ and *D*_1_ stand for the optical density of *β*-actin ladder and sample ladder, respectively. The optical density for each ladder was calculated by the ImageJ software. Data were obtained from three independent experiments. ^∗^*P* < 0.05 versus the 50PD control group.

Compared with the young control cells (30PD), late PD 2BS (50PD) showed a decreased fluorescent intensity of NAO, suggesting a lower mitochondrial mass in 50PD cells. Upon treated with SAL at 10 *μ*M for 48 h, the mitochondrial mass level reflected by the NAO fluorescent intensity in 50PD 2BS was pulled up closed to the young level, which is comparable to that of resveratrol (RES) (Figure 2). Then, the alteration of typical signaling pathways involved in the mitochondrial biogenesis was subsequently considered. As shown in Figure 3, the key regulators of mitochondrial biogenesis including PGC-1*α*, NRF-1, and TFAM were significantly increased in 50PD 2BS incubated with SAL, and a similar effect was observed in resveratrol-treated cells.

### 3.3. Effect of SAL on the Mitochondrial Function in 2BS Fibroblasts

**Figure 4:**
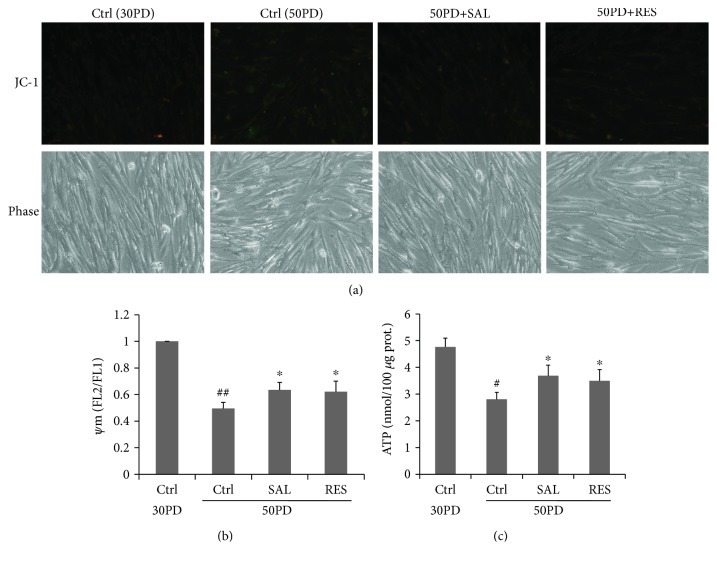
Effect of salidroside (SAL) on the mitochondrial potential (*ΔΨ*m) and ATP production. Cells treated by salidroside (SAL, 10 *μ*M) and resveratrol (RES, 10 *μ*M) for 24 h then were stained by JC-1 for *ΔΨ*m measurement. (a) Representative images of JC-1 staining were acquired by using fluorescence microscopy. (b) Quantitative measurement of *ΔΨ*m was performed by flow cytometric analysis. (c) ATP production was determined by using a commercial kit (ab83355). Cells were treated by salidroside (SAL, 10 *μ*M) and resveratrol (RES, 10 *μ*M) for 24 h and were harvested for ATP production. ^##^*P* < 0.01 versus 30PD control (Ctrl); ^#^*P* < 0.05 versus 30PD control (Ctrl); ^∗^*P* < 0.05 versus control (Ctrl) at 50PD.

Since SAL induced an increment of mitochondrial biogenesis in human fibroblasts, we next asked whether it affects the mitochondrial function. Mitochondrial membrane potential (*ΔΨ*m) and ATP production were used for assessment of a mitochondrial function. In flow cytometric assays of JC-1-stained cells, FL2 (orange) fluorescence reports the J aggregate form of JC-1 which reflects the level of *ΔΨ*m. The FL1 green signal reports the nonaggregate form of the dye and provides a measure to assess variations of dye loading. In untreated young control cells (30PD), flow cytometric analysis revealed a larger population of cells with high *Ψ*m based on FL2/FL1 fluorescence than that of 50PD cells which was partly reversed by SAL and RES treatment (Figure 4(a)). The declined ATP production in late PD cells was also partly reversed by SAL or RES treatment (Figure 4(b)). These results suggest that SAL can rescue the mitochondrial dysfunction via *ΔΨ*m and ATP production in fibroblasts.

### 3.4. SAL Stimulates Mitochondrial Biogenesis Related to the SIRT1 Activation

**Figure 5:**
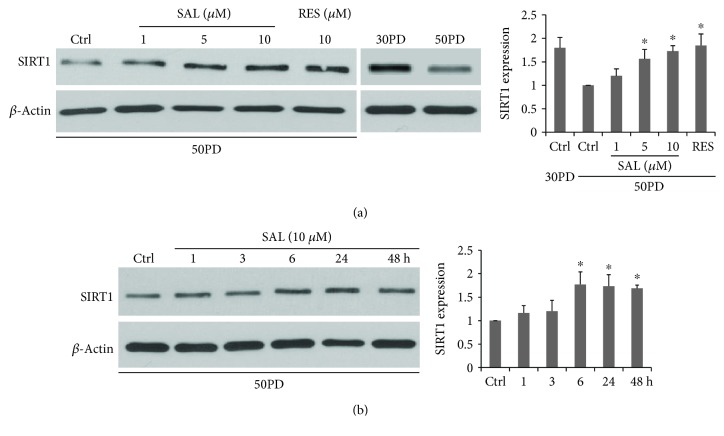
The dose dependence (a) and the time dependence (b) of salidroside (SAL) inducing an increment of SIRT1 expression in 2BS cells. (a) Cells at 50PD were treated by salidroside for 24 h. (b) Cells were exposed to SAL at 10 *μ*M for different times and were harvested for western blot analysis. ^∗^*P* < 0.05 versus control (Ctrl) at 50PD.

**Figure 6:**
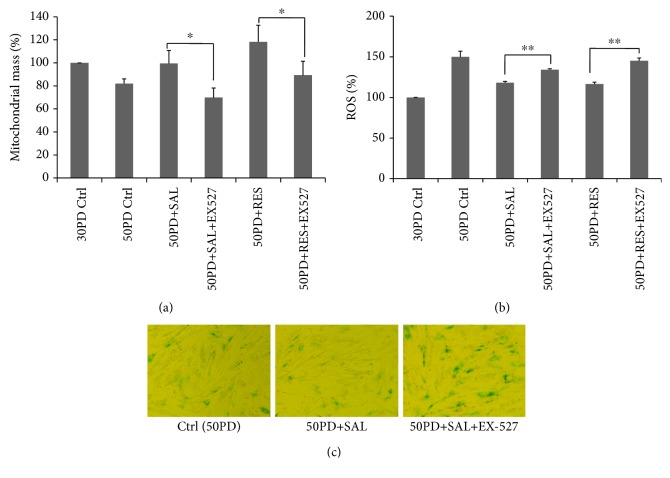
SIRT1 inhibitor EX-527 attenuates salidroside- (SAL-) or resveratrol- (RES-) induced increment of mitochondrial biogenesis (a) or decreased ROS production in 50PD 2BS cells (b) and induces an elevation of SA-*β*-gal activity in SAL-treated 50PD cells (c). EX-527 at 10 *μ*M was added 2 hrs before SAL (10 *μ*M) or RES (10 *μ*M) supplementation in the culture medium and was present for 48 h before harvested. ^∗^*P* < 0.05; ^∗∗^*P* < 0.01.

Since SIRT1 is highly related to mitochondrial biogenesis through activation on PGC1*α* [3], it is interesting to detect the SIRT1 expression. Consistent with previous reports, lower SIRT1 protein expression was observed in late PD fibroblasts in comparison to that of young human fibroblasts [28]. SAL dose dependently increased the SIRT1 protein expression in 50PD 2BS, with 10 *μ*M offering an optimal effect, which was similar to that of resveratrol (Figure 5(a)). For the time course study, SAL started to improve the SIRT1 protein expression at 6 h and maintained this stimulatory effect at least till 48 h (Figure 5(b)). Then, we used a selective SIRT1 inhibitor, Selisistat (EX-27) [29, 30], to test whether SAL-induced mitochondrial biogenesis is dependent on SIRT1 activation. According to the previous studies [30, 31] and our pilot studies, EX-527 at 10 *μ*M was used to inhibit SIRT1 activity and did not cause cell death in 2BS fibroblasts. It was added to the medium 2 h earlier before the SAL or RES treatment and was present until cell harvest. As shown in Figure 6(a), both SAL- and RES-induced increments of mitochondrial mass were suppressed by EX-527 intervention in 50PD cells. Meanwhile, EX-527 pretreatment abolished their inhibition on intracellular ROS production in 50PD 2BS cells (Figure 6(b)). Moreover, an enhanced SA-*β*-gal activity was induced by EX-527 in presenescent cells coincubated with SAL (Figure 6(c)). The above results suggested that inhibition on mitochondrial biogenesis accompanied with an increment of ROS induces cellular senescence rapidly.

### 3.5. Effect of SAL on the miR22 Expression in 2BS Fibroblasts and SAL Partly Rescued Overexpressing miR22-Induced Senescence

**Figure 7:**
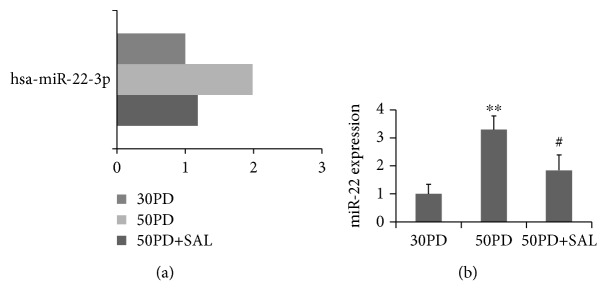
Effect of salidroside (SAL) on the miR-22 expression in near-senescent human fibroblasts. (a) The miR22 expression was analyzed by miRNA microarray, presented as fold changes in the miRNA expression. (b) Relative quantitation of the miR-22 expression in 2BS fibroblasts at 30PD, 50PD, or 50PD cells treated with SAL at 10 *μ*M for 48 h was analyzed by qRT-PCR analysis. miR-22 expression levels were indicated relative to those in 30PD set at 1. U6 was used as an internal normalization control. ^∗∗^*P* < 0.05 versus 30PD; ^#^*P* < 0.05 versus 50PD.

**Figure 8:**
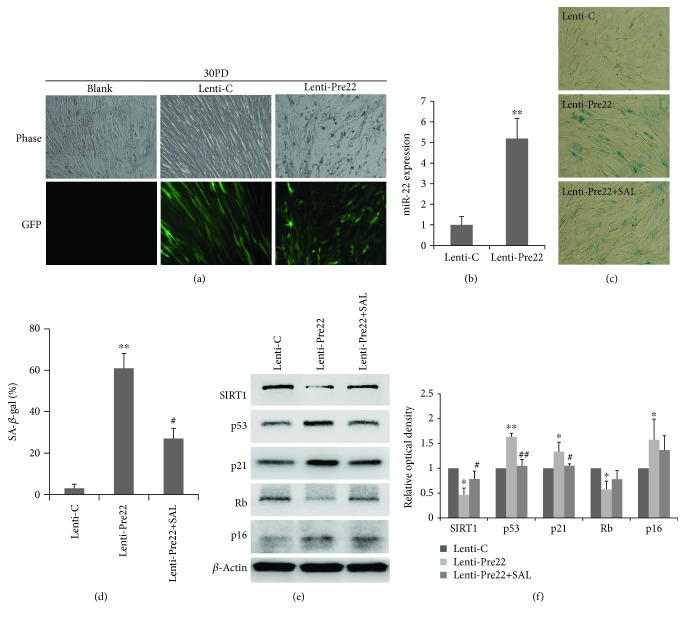
Overexpression of miR22 induces senescence in 30PD 2BS fibroblasts which is partially rescued by salidroside (SAL). (a) Cell morphology was analyzed with fluorescence microscopy at day 3 after infection. GFP-labeled cells indicate infected cells. (b) Relative quantitation of the miR-22 expression in 30PD 2BS cells transfected with Lenti-Pre22 (MOI of 5) was analyzed by qRT-PCR analysis, relative to that in control- (Lenti-C-) transfected cells set at 1. (c) SA-*β*-gal activity was analyzed by phase-contrast microscopy at day 4 after infection with control vector (Lenti-C) or Pre-miR-22 (Lenti-Pre22) and effect of SAL (10 *μ*M) on SA-*β*-gal staining in Lenti-Pre22-transfected 2BS cells at 30PD. (d) The percentage of SA-*β*-gal-positive cells. (e) The protein expression of SIRT1 and cellular senescence molecules including p53, p21, p16, and Rb in 2BS cells transfected with control vector (Lenti-C) or Pre-miR-22 (Lenti-Pre22) and effect of SAL (10 *μ*M) Lenti-Pre22-transfected 2BS cells at 30PD. Representative images were acquired from three repeated experiments. (f) Quantitative analysis of the protein levels of SIRT1, p53, p21, Rb, and p16. ^∗^*P* < 0.05 versus Lenti-C; ^∗∗^*P* < 0.01 versus Lenti-C; ^#^*P* < 0.05 versus Lenti-Pre22; ^##^*P* < 0.01 versus Lenti-Pre22.

To further explore the underlying mechanisms regarding the effect of SAL on delaying cellular senescence, we attempted to screen miRNAs that related to the cellular senescence in human fibroblasts. The change of the miRNA expression profile of 2BS fibroblasts treated by SAL was analyzed by miRNA microarray. Among the miRNAs with obvious alteration upon SAL treatment, miR-22 was increased in near-senescent 2BS cells (50PD) in comparison to that of young cells (30PD), which could be reversed by SAL treatment (Figure 7). The increased miR22 expression in senescent fibroblast was reported in previous studies by using MRC-5 and TIG fibroblastic cell lines, and miR22 was proved to mediate the cellular senescent process through targeting of SIRT1 and CDK6 [19]. To test the effect of miR-22 overexpression on senescence in current 2BS fibroblasts, young cells at 30PD were transfected with a Pre-miR-22 lentiviral (Lenti-Pre22) construct, which stably expresses miR-22 precursor in its native context. Lenti-Pre22-infected cells showed a fivefold increase of mature miR-22 compared with the young control cells (Figures 8(a) and 8(b)) and exhibited an enlarged senescence morphology and SA-*β*-gal-positive staining (Figures 8(c) and 8(d)), accompanied with an increased protein level of senescence-associated molecules p53, p21, and p16 (Figure 8(e)). As anticipated, a decreased SIRT1 protein expression was observed in the Lenti-Pre22-infected cells (Figure 8(e)). However, SAL could partly impede the senescence progression induced by lenti-Pre-miR-22. The increment of SA-*β*-gal activity induced by overexpression of miR-22 was prevented in part by SAL (Figures 8(c) and 8(d)). Besides, declined expression of SIRT1 and increased protein levels of p53, p21, and p16 in Lenti-Pre22-infected cells were partly reversed by SAL treatment (Figure 8(e)). These results implied that SAL stimulates the SIRT1 partly through inhibition on miR-22.

## 4. Discussion

As a key mediator for the benefits of CR, SIRT1 was well accepted as a target for the drug discovery against aging and aging-related degenerative disorders in recent decades. Resveratrol seems to be the first putative activator of SIRT1 [32]. Of all the natural SIRT1 activators discovered to date, RES is still the most potent. Resveratrol was initially identified in 1940 as a phenolic substance in the white hellebore, Veratrum grandiflorum, a flowering plant, and later in grape vines and the Japanese knotweed Polygonum sachalinense [33]. In recent years, SAL was found to exert health benefits such as cognitive improvement and inflammation amelioration probably through modulating SIRT1 [34–36]. However, the relationship of its activation on SIRT1 and replicative senescence still lacks direct evidences, and the detailed mechanism regarding its activation mode on SIRT1 remains elusive. In the current study, our findings suggested that SAL delays replicative senescence in human fibroblasts, which further solidified its antiaging effect based on our previous reports [20–22]. Its stimulatory role on mitochondrial biogenesis through upregulation on SIRT1 in human fibroblasts relates to its efficacy on delaying cellular senescence and antioxidative properties, which revealed a novel mechanistic mode for this naturally occurring compound regarding its beneficial effect against aging. Moreover, SAL delays cellular senescence partly through the miR-22/SIRT1 pathway. MicroRNA-22 (miR-22) has been predicted to potentially target SIRT1, and SAL seems to increase the SIRT1 expression by inhibition on miR-22.

Despite SIRT1, another important factor involved in the regulation of PGC-1*α* transcription is AMP-activated kinase (AMPK). AMPK activity appears to be one of the main factors associated with deficient mitochondrial biogenesis, insulin resistance, and impaired lipid metabolism observed in aged cells [37, 38]. Chronic AMPK inactivation is linked to a marked decrease in mitochondrial biogenesis in aged animals [37]. SAL was reported to exert its benefits through activating AMPK and its related pathways [39, 40]. In the current 2BS fibroblasts, we also found a SAL-induced increase of AMPK*α*-Thr172 phosphorylation, and this activation of AMPK*α* could be blocked by 8-bromo-AMP (8Br-AMP), an AMPK inhibitor [41] ([Supplementary-material supplementary-material-1]). Meanwhile, SAL-induced mitochondrial mass increment and inhibitory on intracellular ROS production in 50PD cells were prevented by 8Br-AMP ([Supplementary-material supplementary-material-1]). Our data suggested that AMPK*α* activation is probably involved in SAL-induced mitochondrial biogenesis in 2BS cells. Furthermore, it was reported that resveratrol directly inhibits cAMP-specific phosphodiesterases (PDE) and identified the cAMP effector protein Epac1 as a key mediator of the effects of resveratrol, which leads to the activation of AMPK and SIRT1 and contributes to the amelioration of aging-related metabolic phenotypes [42]. As a comparable effect of SAL and RES on inducing mitochondrial biogenesis as well as ROS scavenging activity was observed according to our current work, they may share similar molecular mechanisms. Interestingly, according to a RNAseq assay in our recent work [43], SAL was shown to suppress the expression of PDE2A, a member of the PDE super family, which is a dual-substrate PDE towards both cAMP and cGMP. Moreover, according to a clinic trial in human and murine models of diabetes, treatment with a PDE5 inhibitor sildenafil induced increment of SIRT1 through a downregulation of the miR-22 expression, resulting in ameliorating visceral adipose tissue [44]. Thus, for the further detailed mechanisms regarding SAL on the modulation of the miR-22 expression, it seems to be related to the PDE inhibition.

Another important signaling pathway linked to mitochondrial biogenesis is related to eNOS activation [45]. It has been reported that resveratrol stimulates mitochondrial biogenesis in endothelial cells via activation of eNOS [5]. Indeed, a previous study indicated eNOS activation is involved in SAL-induced mitochondrial biogenesis in human umbilical vein endothelial cells (HUVECs) [46]. However, by using a mouse cardiac tissue sample as a positive control for the eNOS immune blotting, our data suggested that the eNOS protein was undetectable in young or near-senescent human 2BS fibroblasts, which were originally isolated from human fetal lung tissues ([Supplementary-material supplementary-material-1]). Consistently, the eNOS inhibitor NG-nitro-L-arginine methyl ester (L-NAME) did not affect the mitochondrial mass either the intracellular ROS production in 2BS cells under SAL or RES treatment in 50PD 2BS ([Supplementary-material supplementary-material-1]). Thus, the mechanisms of SAL on inducing mitochondrial biogenesis seem to be tissue specific. SAL induces mitochondrial biogenesis may be through different signaling pathways in different cell types, and it is a limitation for discovery of agents stimulating mitochondrial biogenesis through activation of eNOS in this cellular senescence model.

As the function of transfected mature miRNA may result from its supraphysiological level, we hereby used a stable miRNA vector that mimics miRNA biological processing. A Pre-miR-22 lentiviral construct (Lenti-Pre22), stably expressing miR-22 precursor in its native context, was used to study the effect of miR-22 on cellular senescence in 2BS fibroblasts. A fivefold increment of mature miR-22 was observed in young 2BS cells at 30PD after transfection with Lenti-Pre22 at MOI of 5, which was a bit higher than its normal level in presenescent 50PD cells (Figure 7) and was similar to the miR-22 level in replicative senescent MRC5 fibroblasts at 58PD reported previously [19]. Thus, Lenti-Pre22 induced cellular senescence in young 2BS cells through upregulation of miR-22, which subsequently suppressed the expression of SIRT1. SAL partly rescued this decline of SIRT1 and impeded the cellular senescence induced by Lenti-pre22. This may be explained due to the multifunction of this natural compound as follows: first, SAL may serve as a direct inhibitor on miR-22 formation, while the detailed mechanisms remain to be further explored. Second, miR-22 induces cellular senescence accompanied with oxidative stress and itself *per se* could mediate the intracellular oxidative stress [47–49], and SAL may directly prevent this process as it is a potent antioxidant according to our previous work and others' [21, 39]. Third, SAL could rescue the declined SIRT1 by other pathways. As described previously, SAL could activate the AMPK which may further stimulate SIRT1 or through a PDE inhibition way [42, 43]. Besides, SAL was reported to increase the expression level of the key protein NAMPT which synthesizes the NAD+, a coenzyme of SIRT1 [50]. In the current work, the enhanced mitochondrial biogenesis induced by SAL seems to be related to the SIRT1 activity, as it was blocked by EX-527, which inhibits SIRT1 activity and hampered the ability of other compounds, e.g., pyrroloquinoline quinone (PQQ), to stimulate mitochondrial biogenesis [29, 51].

## 5. Conclusions

In conclusion, to the best of our knowledge, our work is the first time to investigate the linkage of the natural occurring compound, salidroside, regarding its interventional effect on cellular senescence via modulation on the mitochondrial biogenesis and its regulation on microRNAs, specifically through the miR-22/SIRT1 pathway. Our findings further pave the way for the utilization of salidroside for delaying aging as well as for its clinical application against aging-related degenerative disorders in the future.

## Figures and Tables

**Table 1:** The effect of salidroside on the life spans of 2BS cells in CPDs.

Group	Treatment	Time of transfer to a special medium	*n*	CPDs	Average PDs per week
I	Control	—	3	55.3 ± 3.7	1.6 ± 0.1
II	SAL (5 *μ*M)	30PD	3	62.8 ± 4.1	2.1 ± 0.2^∗^
III	SAL (10 *μ*M)	30PD	3	63.5 ± 5.3	2.2 ± 0.2^∗^

*Note.* Human fibroblast 2BS cells at 30PD were cultured in DMEM supplemented with salidroside (SAL) at 5 *μ*M and 10 *μ*M, respectively. When the confluence of the culture was reached 70-80%, the cells were split in ratios of 1 : 2 or 1 : 4 and were continually cultured in the indicated medium. Control cells were cultured in a 0.01% supplemented medium. CPDs (cumulative population doublings) were calculated as log2 (*D*/*D*_0_), where *D* and *D*_0_ were defined as the density of cells at the time of harvesting and seeding, respectively. The last culture was defined as the subculture that could not be confluent in 15 days. Data were obtained from three independent experiments (^∗^*P* < 0.05 vs. *I*).

## Data Availability

The data used to support the findings of this study are available from the corresponding authors upon request.

## Supplementary Materials

Supplementary Materials The figure legend for the two figures are as follows: Figure S1: AMPK inhibitor 8-bromo-AMP (8Br-AMP) blocks salidroside- (SAL-) induced increased AMPK*α*-Thr172 phosphorylation (A) and abolished salidroside- (SAL-) or resveratrol- (RES-) induced increment of mitochondrial biogenesis (B) or decreased ROS production in 50PD 2BS cells (C). 8Br-AMP at 50 *μ*M was added 2 h before SAL (10 *μ*M) or RES (10 *μ*M) supplementation in the culture medium and was present for 48 h before harvested. Figure S2: the protein expression of eNOS is undetectable in young 30PD or near-senescent 50PD 2BS fibroblasts when compared to an eNOS-positive expression sample, mouse cardiac tissue homogenate (A). The eNOS inhibitor NG-nitro-L-arginine methyl ester (L-NAME) does not influence the increment of mitochondrial biogenesis (B) or decreased ROS production induced by salidroside (SAL) or resveratrol (RES) in 50PD 2BS cells (C). L-NAME at 200 *μ*M was added 2 h before SAL (10 *μ*M) or RES (10 *μ*M) supplementation in the culture medium and was present for 48 h before harvested.
