# Supplementary material for: Salidroside Delays Cellular Senescence by Stimulating Mitochondrial Biogenesis Partly through a miR-22/SIRT-1 Pathway
Source: Oxid Med Cell Longev. 2019 Sep 12;2019:5276096. doi: 10.1155/2019/5276096 (PMC6757293; doi:10.1155/2019/5276096)
Supplement: Supplementary Materials — The figure legend for the two figures are as follows: Figure S1: AMPK inhibitor 8-bromo-AMP (8Br-AMP) blocks salidroside- (SAL-) induced increased AMPKα-Thr172 phosphorylation (A) and abolished salidroside- (SAL-) or resveratrol- (RES-) induced increment of mitochondrial biogenesis (B) or decreased ROS production in 50PD 2BS cells (C). 8Br-AMP at 50 μM was added 2 h before SAL (10 μM) or RES (10 μM) supplementation in the culture medium and was present for 48 h before harvested. Figure S2: the protein expression of eNOS is undetectable in young 30PD or near-senescent 50PD 2BS fibroblasts when compared to an eNOS-positive expression sample, mouse cardiac tissue homogenate (A). The eNOS inhibitor NG-nitro-L-arginine methyl ester (L-NAME) does not influence the increment of mitochondrial biogenesis (B) or decreased ROS production induced by salidroside (SAL) or resveratrol (RES) in 50PD 2BS cells (C). L-NAME at 200 μM was added 2 h before SAL (10 μM) or RES (10 μM) supplementation in the culture medium and was present for 48 h before harvested. [file 5276096.f1.pdf]

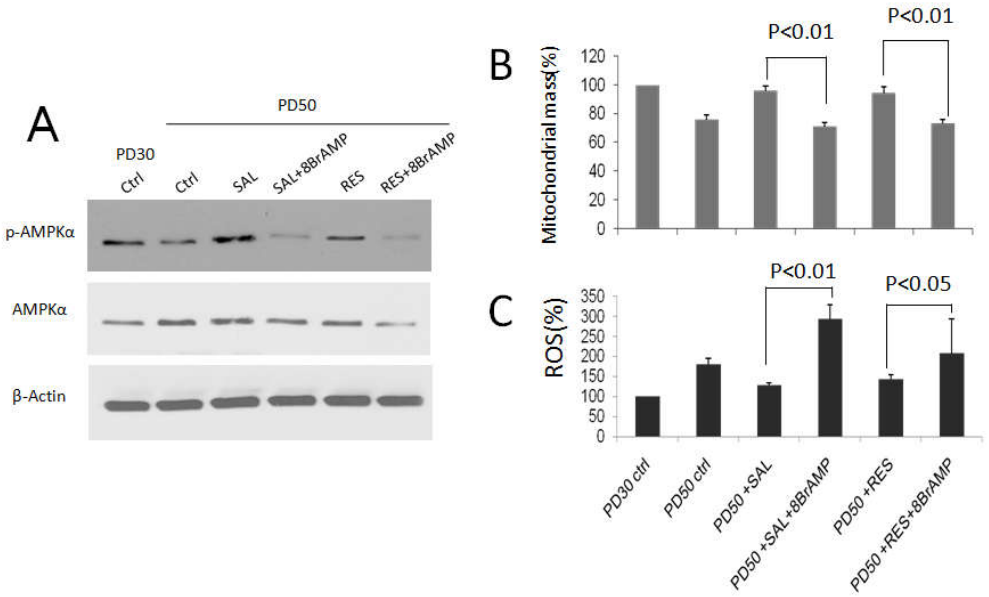

FIGURE S1: AMPK inhibitor 8-bromo-AMP (8Br-AMP) blocks salidroside (SAL) induced increased AMPKα-Thr172 phosphorylation (A) and abolished salidroside (SAL) or resveratrol (RES) induced increment of mitochondrial biogenesis (B) or decreased ROS production in PD50 2BS cells (C). 8Br-AMP at 50μM were added 2 h before SAL (10 μM) or RES (10 μM) supplementation in the culture medium, and was present for 48h before harvested.

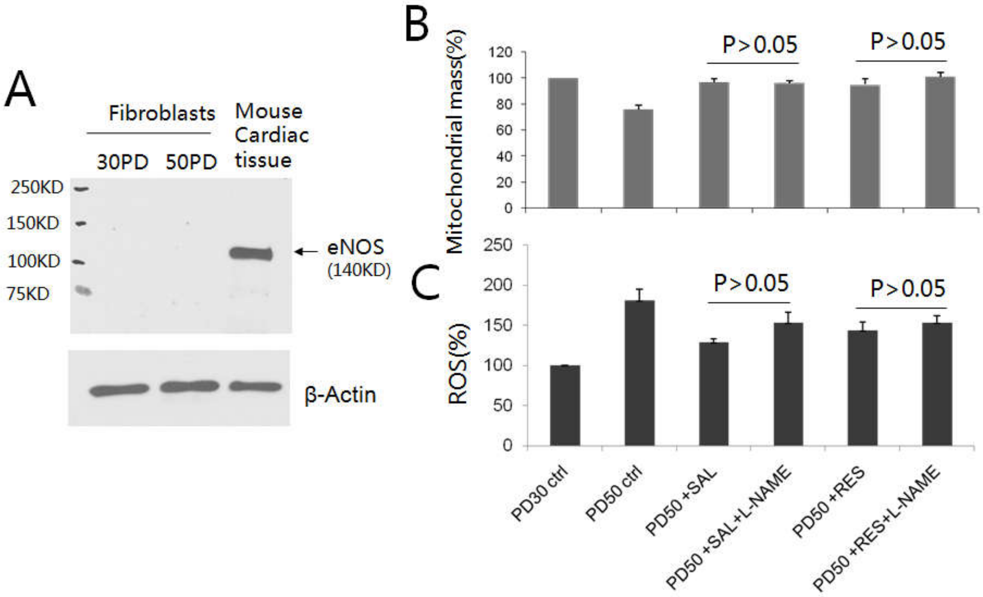

FIGURE S2: The protein expression of eNOS is undetectable in young PD30 or near-senescent PD50 2BS fibroblasts when compared to an eNOS positive expression sample, mouse cardiac tissue homogenate (A). The eNOS inhibitor NG-nitro-L-arginine methyl ester (L-NAME) does not influence the increment of mitochondrial biogenesis (B) or decreased ROS production induced by salidroside (SAL) or resveratrol (RES) in PD50 2BS cells (C). L-NAME at 200μM were added 2 h before SAL (10 μM) or RES (10 μM) supplementation in the culture medium, and was present for 48h before harvested.
